# Prying reduction with mosquito forceps versus limited open reduction for irreducible distal radius‐ulna fractures in older children: a retrospective study

**DOI:** 10.1186/s12891-021-04024-y

**Published:** 2021-02-05

**Authors:** Qiang Huang, Fei Su, Zhi Meng Wang, Han Zhong Xue, Liang Sun, Teng Ma, Qian Wang, Yao Lu, Ming Li, Cheng Ren, Cong Ming Zhang, Kun Zhang, Zhong Li

**Affiliations:** Xi’an Hong Hui hospital, Xi’an Jiaotong University College of Medicine, 710000 Xi’an, China

**Keywords:** Prying reduction, Mosquito forceps, Distal radius-ulna fracture, Irreducible, Older children

## Abstract

**Background:**

There are disputes about which reduction technique should be adopted in treatment of distal radius-ulna fractures in older children who failed to achieve manual reduction. This study compared clinical effects between prying reduction with mosquito forceps (PRMF) and limited open reduction (LOR) of treating irreducible distal radius-ulna fractures in older children.

**Methods:**

One hundred ten children with irreducible distal radius-ulna fractures were selected from January 2015 to December 2017 in Xi’an Hong Hui hospital. Retrospective analysis was performed. According to different reduction techniques, these children were divided into PRMF group (59 cases) and LOR group (51 cases). All children were treated with percutaneous Kirschner wire fixation and external fixation with plaster. Operation indexes, complications and wrist joint functions were compared between the two groups.

**Results:**

Operation time of PRMF group was shorter than that of LOR group (*P <* 0.05). Incision length in PRMF group was less than that in LOR group (*P <* 0.05). Bleeding volume of PRMF group was less than that of LOR group (*P <* 0.05). Incidence of complications in PRMF group was lower than that in LOR group.

**Conclusions:**

Compared with limited open reduction, it has better clinical effects of prying reduction with mosquito forceps in treatment of irreducible distal radius-ulna fractures in older children. This technique has the advantages of simple operation, less trauma, less bleeding and fewer complications, which is worthy of clinical promotion.

## Background

Distal radius-ulna fractures of children are one of the most common fracture types in children. According to literatures, distal radius-ulna fractures in children account for 31% of all fractures in children [1, 2]. The average age of boys with this fracture is 10.4 years old while 9.3 years old among girls [1, 2]. Distal radius-ulna fractures are common in sports injuries [1]. Children with distal radius-ulna fractures are mostly caused by indirect violence, and also be caused by direct violence on distal forearm. When the wrist joint is in dorsal extension position, the palm encounters violence, resulting in an extension fracture. The distal fragment often displaces to dorsal side. While the wrist joint encounters violence during metacarpal flexion, a flexion fracture occurs, and the distal fragment shifts to the palmar side. Standard treatment is closed reduction and plaster fixation in a distal radius-ulna fracture [3–5]. Most children can obtain satisfactory results after conservative treatment because of their strong ability of bone shaping. Although the effect of conservative treatment is exact, there are some risks, such as difficult reduction, long time of plaster external fixation, and redisplacement of fractures [6, 7]. What’s more, some children have serious initial dislocations, soft tissue insertions and so on, which lead to the failure of closed reduction [6–8]. Repeated closed reduction will aggravate soft tissue injuries, even lead to nerve and vascular stretch injuries, resulting in serious complications. Usually, when 3 times of closed reduction fails, prying reduction or open reduction is needed. The authors used mosquito forceps for prying reduction and achieved satisfactory results. It is reported as follows.

## Patients and methods

Inclusion criteria: (1) Children aged 8–15 years; (2) Children with only distal radius-ulna fracture; (3) Children with only metaphyseal fracture; (4) According to AO/ASIF classification, children were classified as A1 and A2 fractures; (5) Children could be followed up completely. Exclusion criteria: (1) Children younger than 8 years or older than 15 years; (2) Children with multiple fractures; (3) Children achieved closed reduction successfully; (4) Children with open fractures; (5) Children need surgical exploration because of neurovascular injuries; (6) Children with incomplete clinical data.

General information: 110 children with irreducible distal radius-ulna fractures were selected from January 2015 to December 2017 in Xi’an Hong Hui hospital. All patients were treated with percutaneous Kirschner wire fixation and external fixation with plaster. There were 60 boys and 50 girls, aged between 8 and 15 years. According to AO/ASIF classification, all children were classified as A1 and A2 fractures. 59 cases were treated by PRMF technique while 51 cases by LOR technique. There was no significant difference in age, gender and fracture type between the two groups (*P >* 0.05, Table 1).

**Table 1:** Demographics of children with distal radius-ulna fractures treated by PRMF and LOR

Group	n	Age (x̅ ±s,year)	Gender		AO/ASIF Typing	
			male	female	A1	A2
PRMF	59	11.9 ± 2.3	34	25	32	27
LOR	51	12.2 ± 1.8	30	21	29	22
t value		0.77				
χ^2^ value			0.02		0.08	
*P* value		> 0.05	> 0.05		> 0.05	

PRMF stands for prying reduction with mosquito forceps, LOR stands for limited open reduction

### Methods

All children were given routine examinations after admission. During physical examination, attention should be paid to the condition of nerves and blood vessels and the presence of osteofascial compartment syndrome. Routine X-ray films were taken, including anteroposterior and lateral view of wrist joint and full length of ulna and radius. Children were treated with detumescence, elevation of affected forearm and temporary fixation of plaster before operation. Parents or guardians of children signed the informed consent before operation.

### Procedure of PRMF group

**Fig. 1:**
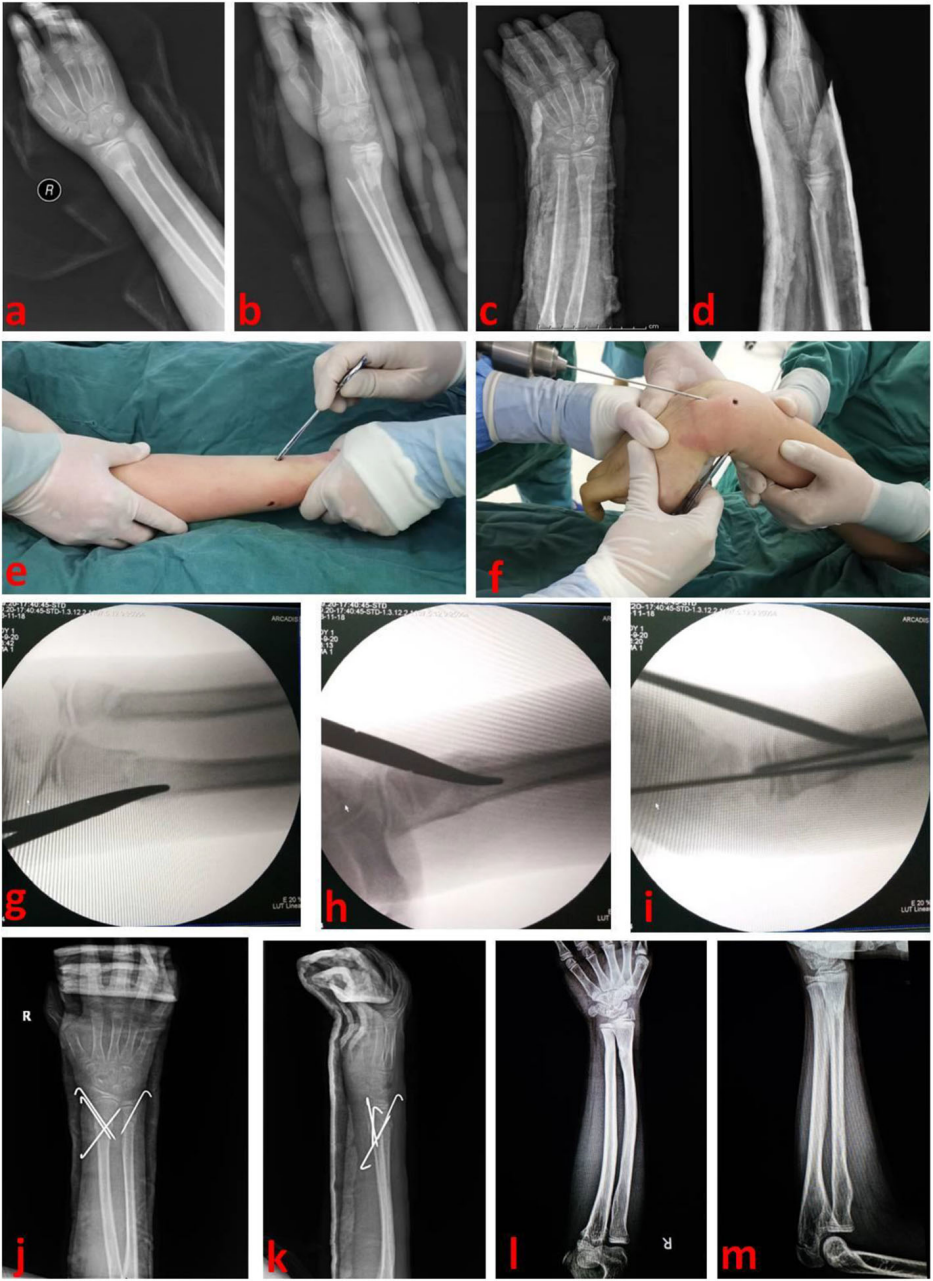
An 11-year-old child suffered from distal radius-ulna fracture and was reduced by PRMF technique. a and b: Preoperative X-ray films showed distal radius-ulna fracture of right forearm; c and d: There was still obvious fracture displacement after manual reduction and plaster fixation; e: Intra-operative prying and manual reduction; f: During the operation, reduction was maintained and Kirschner wire fixation was performed; g, h and i: X-ray films showed that, PRMF technique was used for reduction and maintaining reduction; j and k: X-ray films after the operation showed that this child achieved good reduction and percutaneous Kirschner wire fixation; l and m: The distal radius-ulna fracture healed well and X-ray films were taken after Kirschner wires were removed. PRMF stands for prying reduction with mosquito forceps

After failure of closed reduction, the operator decided to use PRMF technique. The child was given general anesthesia and routine disinfection. Position of fracture site was determined by fluoroscopy. The radius was reduced first. According to displacement of the fracture, prying reduction was performed from palmar or dorsal side. A 0.5 cm incision was taken at the fracture site, and soft tissues were carefully separated. The incision was located on the radial side of the distal forearm via ventral approach. After the skin was cut, it was separated longitudinally with mosquito forceps. Pay attention to avoid lateral separation. It usually entered from the flexor carpi radialis and the radial artery space. In the dorsal approach, it entered from the tendon space. The surgeon inserted mosquito forceps into the above incision and explored the fracture surface. The blood and hematoma were drained from the minimal wound. The mosquito forceps were extended into the fracture space and played the role of prying or jacking according to the fracture displacement. Usually, the surgeon gently pryed the fracture fragment with another fragment as the fulcrum. Prying reduction was often combined with manual reduction. The assistant should pull the proximal and distal part to assist in reduction. Most distal radius-ulna fractures have caniniform insertion and overlapping displacement. Satisfactory reduction can be achieved by prying and pulling. When there is soft tissue insertion at the fracture site, mosquito forceps can be used to release soft tissue insertion, and then displacement can be reduced by prying and manual reduction. Reduction was maintained by the operator, and the fracture was cross fixed by the assistant through Kirschner wires. After achieving satisfactory reduction of the radius, the ulna can usually be reduced by itself. If distal ulna fracture was still in poor position, the same manipulations could be used for prying reduction, and Kirschner wire fixation should be selected according to the stability of ulna fracture. After confirming that reduction and fixation were satisfactory by fluoroscopy, Kirschner wires were bent and kept outside the skin. Finally, the forearm was placed in supination position, and a short arm plaster was externally fixed. Distal end of the plaster was to the metacarpophalangeal joint and proximal end to the elbow joint. A typical case is shown in Fig. 1.

### Procedure of LOR group

After failure of closed reduction, the operator decided to adopt LOR technique. According to displacement of the fracture, operators could choose palmar Henry approach or radial dorsal approach. The incision length was about 3–5 cm. During the volar incision, the fracture site was exposed from the space between radial artery and flexor carpi radialis. When a dorsal incision was made, the fracture site was exposed from the space between the extensor tendons. Attention should be paid not to peel off the periosteum too much, and not to peel off again when it was close to the epiphysis. Hematoma was cleared at the fracture site and soft tissues were released. Under direct vision, fragments were reduced by prying, forceps and manipulative reduction. Percutaneous cross Kirschner wires were used to fix the distal radius fracture. Length of Kirschner wires was confirmed by fluoroscopy. When the radius was reduced and fixed well, reduction of distal ulna was evaluated. Kirschner wires were bent and kept outside the skin. Finally, it was performed of external fixation with plaster.

#### Postoperative management

After 2 weeks, the plaster was changed to a suitable brace, and the brace could be removed intermittently for wrist function exercise. The brace was used to assist fixation for another 1–2 weeks. X-ray of wrist joint was reexamined every 2 weeks to evaluate fracture healing. Kirschner wires were removed after 4–6 weeks of initial healing.

#### Observation indexes

It was compared between the two groups of incision length, bleeding volume, intra-operative fluoroscopy times and operation time. Complications were observed. Children were followed up for 1 year. At 1 year after operation, visual analog scale (VAS) score was used to compare the degree of wrist pain between the two groups. Palmar tilting angle and ulna deviation angle were compared of the two groups. Gartland werley [9] score was used to evaluate wrist function from subjective (activity limitation, pain, dysfunction, etc) and objective (range of motion, neuromuscular evaluation, grip strength, etc) evaluation. 0–2 points were defined as excellent, 3–8 points were good, 9–14 points were fair, and ≥ 15 points were poor.

### Statistical methods

SPSS 23.0 software was used to process the data and measurement data were expressed as the mean ± standard deviation. The unpaired t test was used for comparisons between the two groups, including age, operation index and wrist function score. Count data were analyzed using the χ2 test, including gender and fracture classification. *P* < 0.05 was considered to be statistically significant.

## Results

### Operation indexes

The incision length of PRMF group was 0.5 ± 0.2 cm, which was smaller than LOR group of 4.2 ± 1.3 cm (*P* < 0.05). The operation time of PRMF group was 25.4 ± 4.8 min, shorter than that of 36.8 ± 5.5 min in LOR group (*P* < 0.05). The bleeding volume of PRMF group was 5.8 ± 2.5 ml, less than LOR group of 20.6 ± 3.4 ml. The fluoroscopy times of PRMF group were 8.4 ± 1.5, which were more than LOR group of 4.7 ± 2.1 (P < 0.05). The above results are shown in Table 2.

**Table 2:** Operation indexes of PRMF and LOR in treating distal radius-ulna fractures

Group	Cases (n)	Incision length (cm)	Operation time(min)	fluoroscopy times (n)	Bleeding volume (ml)
PRMF	59	0.5 ± 0.2	25.4 ± 4.8	8.4 ± 1.5	5.8 ± 2.5
LOR	51	4.2 ± 1.3	36.8 ± 5.5	4.7 ± 2.1	20.6 ± 3.4
t value		20.12	11.49	10.48	25.66
P value		< 0.05	< 0.05	< 0.05	< 0.05

### Wrist function scores

At 1 year after operation, both groups achieved good wrist joint functions. X-ray showed that fracture fragments were well aligned and fractures healed. Palmar tilting angle and ulna deviation angle were basically normal. Movement of wrist joint (ulna deviation, radial deviation, palmar flexion, dorsal extension and forearm rotation) returned to generally normal. The VAS score of wrist joint was 1.3 ± 0.6 in PRMF group and 1.2 ± 0.8 in LOR group (*P* > 0.05, Table 3). Moreover, the Gartland werley score was 2.0 ± 1.5 in PRMF group and 2.1 ± 1.7 in LOR group (P > 0.05, Table 3).

**Table 3:** Clinical efficacy of PRMF and LOR in treating distal radius-ulna fractures

Group	Cases (n)	Palmar tilting angle (°)	Ulna deviation angle (°)	VAS score	Gartland-Werley score
PRMF	59	10.3 ± 1.8	20.6 ± 2.5	1.3 ± 0.6	2.0 ± 1.5
LOR	51	9.9 ± 1.3	20.1 ± 1.8	1.2 ± 0.8	2.1 ± 1.7
t value		1.35	1.21	0.73	0.32
P value		> 0.05	> 0.05	> 0.05	> 0.05

VAS stands for visual analog scale

### Complications

The incidence of common complications in PRMF group was lower than that in LOR group, including incision infection, skin irritation and nerve injury (Table 4). No serious complications occurred in both groups, such as compartment syndrome and growth arrest.

**Table 4:** Complications of PRMF and LOR in treating distal radius-ulna fractures in older children

Group	Incision infection n(%)	Skin irritation n(%)	Nerve injury n(%)	Severe complications n(%)
PRMF	0 (0%)	2 (3.4%)	0 (0%)	0 (0%)
LOR	2 (3.9%)	3 (5.9%)	2 (3.9%)	0 (0%)

## Discussion and conclusions

Distal part of ulna and radius is the junction of dense bone and cancellous bone. Cortical bone becomes thinner in this area. There is a lack of compact and regular bone plates and units. Epiphysis exists at the distal part of radius and ulna in children, where the growth rate exceeds the calcification of bone, resulting in relatively low bone mineral density. Therefore, distal part of ulna and radius is an anatomic weak area, which is prone to fracture. Distal radius-ulna fractures are common in children, and most of them are closed injuries [1, 2]. The most common fracture types are palmar dorsum overlapping displacement. It is rare of long oblique type, spiral type and comminuted type.

Children under 8 years old after fracture have a strong shaping ability [10]. Most of the children in this age group can be treated conservatively [11, 12]. The molding time can be as long as several months. Older children may have incomplete shaping and even residual local deformities [13, 14]. The conservative treatment is usually closed reduction and plaster external fixation. Before reduction, operators should be clear with the strength, direction of external force and the traction effect of osteofascial and muscle on the fracture fragment. After understanding the mechanism of injury, fracture displacement is reduced by reversing injury mechanism. Most of the children can achieve satisfactory reduction by simple traction and manual reduction [3–5]. However, during the period of plaster fixation, due to the regression of forearm swelling and plaster loosening, about one third of children with fracture redisplacement were reported in literatures [15–17]. If redisplacement of the fracture is not serious, it can be untreated. However, if redisplacement of the fracture is significant, it needs to be reduced again. Due to serrated fracture site in some children, length of the distal part is small and direct traction is weak, which makes it difficult to remove the insertion. At this time, satisfactory reduction can not be achieved by only closed reduction. Forced closed reduction can aggravate soft tissue injuries, lead to neurovascular stretch injuries, and even lead to osteofascial compartment syndrome, epiphyseal injuries and other serious complications [18]. Most fractures of distal radius-ulna are straight fractures in children. The distal fragment displaces to the dorsal side. Most of the dorsal periosteum is intact, the broken sites are not easy to pull apart, and the fracture sites are still overlapped. Sometimes soft tissues or periosteum are inserted at the fracture site, which will prevent fracture reduction. In addition, some children struggle and do not cooperate in the process of reduction, which brings difficulties to manual reduction. All of these reasons will lead to the failure of closed reduction. When manual closed reduction fails, scholars still have disputes about which technique to use for reduction.

Operators may solve the problem of irreducible distal radius-ulna fractures by open reduction technique. According to displacement of the fracture, incision can be made on the palmar or dorsal side. However, open reduction is traumatic, bleeding, leaving surgical scars, and even a certain probability of damaging epiphysis, resulting in growth arrest and other serious complications. Some scholars used minimally invasive prying reduction technique of Kirschner wires in the treatment of irreducible distal radius fractures in children [19, 20]. Pavone et al. reported the midterm results of surgical treatment of displaced proximal humeral fractures in children [21]. They showed excellent results with a minimally invasive treatment with percutaneous k-wires. The excellent outcomes they observed led them to prefer the mini-invasive surgical approach in NH grade III fractures [21]. As a reduction tool, Kirschner wire is usually inserted at the fracture site, which works through the lever principle. However, a fine Kirschner wire is easy to bend, and the force may be insufficient when prying through the lever principle. When a thick Kirschner wire is used for prying, its sharp tip may aggravate injury of soft tissues, and even cause nerve and blood vessel injuries. When soft tissues are inserted at the fracture site, it is necessary to release soft tissues, and at this time, Kirschner wire can not play a good role. So, is there a more suitable tool to achieve the purpose of prying reduction?

The authors used mosquito forceps in older children with distal radius-ulna fractures, and achieved satisfactory reduction effects. Mosquito forceps are small and fine, with blunt tips, and are always available in the operating room. This design allows it to be inserted through a minimally invasive incision. Mosquito forceps can provide enough strength during prying reduction without increasing the risk of nerve and vascular injuries. Therefore, mosquito forceps can be a good tool for prying reduction. The authors’ retrospective study shows that, compared with limited open reduction, it has shorter incision length, less bleeding and shorter operation time of prying reduction with mosquito forceps. Due to the minimally invasive incision, only 0.5 cm, children feel mild pain postoperatively. A smaller incision means less damage to soft tissues and blood supplies, so that there is less bleeding during the operation process, and the incision is easier to heal. It can significantly shorten the hospitalization time of children after operation, which is also in line with the concept of accelerating rehabilitation. However, the fluoroscopy times were slightly increased during prying reduction. Clinicians should pay attention to radiation protection. Although both groups achieved good wrist functions, incidence of common postoperative complications was lower in PRMF group. Importantly, there are some pitfalls of PRMF technique that surgeon should avoid. Firstly, pay attention to gentle prying, do not break the fracture fragments. Secondly, in the process of prying, the two teeth of mosquito forceps should be closed to avoid clamping nerves and blood vessels. Thirdly, when the assistant assists through longitudinal traction, pay attention to avoid soft tissue traction injury. Fourthly, do not use the tip of mosquito forceps to probe the distal part of the fracture fragment excessively, so as to avoid damaging the epiphysis.

There are still some limitations in this study. The number of children included in this study was small, and the follow-up time was short. This study was limited to the metaphysis of the distal radius and ulna, and did not include children with epiphyseal fractures. Sometimes, metaphyseal fracture and epiphyseal fracture can occur in one child at the same time. In addition, this study was a single center clinical study. In the future, the authors will further carry out multi-center clinical research. The above deficiencies will be corrected in further research.

## Conclusions

Satisfactory reduction can be achieved by both prying reduction with mosquito forceps and limited open reduction in older children with irreducible distal radius-ulna fractures. Compared with limited open reduction, it has better clinical effects of prying reduction with mosquito forceps. It has the advantages of simple operation, less trauma, less bleeding and fewer complications, which is worthy of clinical promotion.

## Data Availability

The data and materials are available from the corresponding author on reasonable request.

## Abbreviations

PRMF: prying reduction with mosquito forceps
LOR: limited open reduction
VAS: visual analog scale

## References

[1] Randsborg PH, Gulbrandsen P, Benth JS, Sivertsen EA, Hammer OL, Fuglesang HFS, Årøen A (2013). Fractures in children: epidemiology and activity-specific fracture rates. J Bone Joint Surg Am.

[2] Hedstrom EM, Svensson O, Bergstrom U, Michno P (2010). Epidemiology of fractures in children and adolescents. Acta Orthop.

[3] Pannu GS, Herman M (2015). Distal radius-ulna fractures in children. Orthop Clin N Am.

[4] Davidson JS, Brown DJ, Barnes SN, Bruce CE (2001). Simple treatment for torus fractures of the distal radius. J Bone Joint Surg Br..

[5] Crawford SN, Lee LS, Izuka BH (2012). Closed treatment of overriding distal radial fractures without reduction in children. J Bone Joint Surg Am.

[6] Zamzam MM, Khoshhal KI (2005). Displaced fracture of the distal radius in children: factors responsible for redisplacement after closed reduction. J Bone Joint Surg Br..

[7] Mani GV, Hui PW, Cheng JC (1993). Translation of the radius as a predictor of outcome in distal radial fractures of children. J Bone Joint Surg Br.

[8] Asadollahi S, Ooi KS, Hau RC (2015). Distal radial fractures in children: risk factors for redisplacement following closed reduction. J Pediatr Orthop.

[9] Gartland JJ, Werley CW (1951). Evaluation of healed Colles’ fractures. J Bone Joint Surg Am.

[10] Schmittenbecher PP (2005). State-of-the-art treatment of forearm shaft fractures. Injury..

[11] Alemdaroglu KB, Iltar S, Cimen O, Uysal M, Atlihan D (2008). Risk factors in redisplacement of distal radial fractures in children. J Bone Joint Surg Am.

[12] McLauchlan GJ, Cowan B, Annan IH, Robb JE (2002). Management of completely displaced metaphyseal fractures of the distal radius in children-a prospective, randomised controlled trial. J Bone Joint Surg Br..

[13] Hove LM, Brudvik C (2008). Displaced paediatric fractures of the distal radius. Arch Orthop Trauma Surg.

[14] Lefevre Y, Laville JM, Boullet F, Salmeron F (2012). Early correction of paediatric malunited distal metaphyseal radius fractures using percutaneous callus osteoclasis (“calloclasis”). Orthop Traumatol Surg Res.

[15] Fenton P, Nightingale P, Hodson J, Jonathan L (2012). Factors in redisplacement of paediatric distal radius fractures. J Pediatr Orthop B..

[16] Devalia KL, Asaad SS, Kakkar R (2011). Risk of redisplacement after first successful reduction in paediatric distal radius fractures: sensitivity assessment of casting indices. J Pediatr Orthop B.

[17] Jordan RW, Westacott DJ (2012). Displaced pediatric distal radius fractures—when should we use percutaneous wires?. Injury..

[18] Abzug JM, Little K, Kozin SH (2014). Physeal arrest of the distal radius. J Am Acad Orthop Surg.

[19] Satish BR, Vinodkumar M, Suresh M, Prasad YS, Krishnaraj J (2014). Closed reduction and K-wiring with the Kapandji technique for completely displaced pediatric distal radial fractures. Orthopedics..

[20] Parikh SN, Jain VV, Youngquist J (2013). Intrafocal pinning for distal radius metaphyseal fractures in children. Orthopedics..

[21] Pavone V, Claudia de Cristo C, Cannavo` L, Gianluca Testa G, Buscema A, Condorelli G, Sessa G (2016). Midterm results of surgical treatment of displaced proximal humeral fractures in children. Eur J Orthop Surg Traumatol.

